# Molecular epidemiology of string test-positive Klebsiella pneumoniae isolates in Huzhou, China, 2020-2023

**DOI:** 10.3389/fcimb.2024.1411658

**Published:** 2024-08-06

**Authors:** Wei Yan, Deshun Xu, Yuehua Shen, Fenfen Dong, Lei Ji

**Affiliations:** Microbe Laboratory, Huzhou Center for Disease Control and Prevention, Huzhou, Zhejiang, China

**Keywords:** string test-positive KP, hypermucoviscosity, virulence, whole genome sequencing, molecular epidemiology

## Abstract

**Objective:**

This study used whole-genome sequencing (WGS) to explore the genetic diversity, virulence factors, and antimicrobial resistance determinants of string test-positive *Klebsiella pneumoniae* (KP) over a 4-year surveillance period in Huzhou, China.

**Methods:**

In total, 632 clinical isolates were collected via hospital surveillance from 2020 to 2023; 100 were positive in the string test and these 100 strains were subjected to antimicrobial susceptibility testing using an agar dilution method followed by WGS.

**Results:**

The resistance rates to cefotaxime (77.0%), trimethoprim-sulfamethoxazole (67.0%), and nalidixic acid (64.0%) were high. Multilocus sequence typing revealed high genetic diversity; there were 33 sequence types (STs) and 15 capsular serotypes. The most common ST was ST23 (16.0%) and the most common capsular serotype was K1 (22.5%). Virulome analysis revealed among-strain differences in virulence factors that affected bacterial adherence, efflux pump action, iron uptake, nutritional factors, metabolic regulation, the secretion system, and toxin production. The Kleborate strain-specific virulence scores of all 100 string test-positive KPs were derived: 28 strains scored 5, 28 scored 4, 21 scored 3, 12 scored 1, and 11 scored 0. All 77 strains with scores of 3 to 5 contained the *iucA* gene. The phylogeny based on whole-genome single nucleotide polymorphisms (wgSNPs) indicated high clonality; the string test-positive KP strains were grouped into six clades. Closely related isolates in each genetic cluster usually shared STs.

**Conclusion:**

The present study highlights the significance of the KP *iuc*A gene in terms of hypervirulence and the diverse genotypes of string test-positive KP strains isolated in Huzhou hospitals.

## Introduction

*Klebsiella pneumoniae* (KP) is an increasingly significant pathogen associated with severe life-threatening diseases of various organs (Russo and Marr, 2019). It is an opportunistic Gram-negative pathogen that causes both nosocomial and community-acquired infections (Choby et al., 2020) including pneumonia, bacteremia, urinary tract infections, and others (Fazili et al., 2016). Hypervirulent KP (hvKP) is linked to an “invasive syndrome” that was first identified in Taiwanese patients with liver abscesses in 1986; hvKP is more virulent than “classical KP” (cKP) (Liu et al., 1986). Unlike cKP, hvKP exhibits hypermucoviscosity on blood plates. At the molecular level, the unique hvKP virulence genes promote both invasion and transmission, associated with multi-site and secondary metastatic infections. HvKP infection is linked to severe pathogenicity and high mortality (Shon et al., 2013; Fazili et al., 2016).

As sequencing costs decrease, bacterial whole-genome sequencing (WGS) has become more common. WGS rapidly generates accurate data that aid typing, phylogenetic analyses, and exploration of bacterial virulence and resistance characteristics (Ribot et al., 2019). WGS is increasingly used by public health laboratories that engage in pathogen surveillance; it is replacing conventional technologies.

The definition of hvKP is based on a combination of clinical and microbiological characteristics that differ from those of cKP (Harada and Doi, 2018). Not all hvKP strains are hypermucoviscous; certain cKP strains exhibit this feature (Russo and Marr, 2019). Hypermucoviscosity (associated with hypervirulence) reflects overexpression of capsular polysaccharides (Anantharajah et al., 2022). Experimentally, *iuc* and/or *rmpA* or *rmpA2* are the best hypervirulence markers; if they are absent, hypervirulence is lacking (Russo and Marr, 2019).

String test-positivity indicates hypervirulence. This study collected 632 KP strains from sentinel Huzhou hospitals from 2020 to 2023. In total, 100 hypermucoviscous strains were identified using the string test and subjected to antimicrobial susceptibility testing (AST) and WGS. Antimicrobial resistance (AMR) profiles, capsular serotypes, sequence types, antimicrobial resistance and virulence genes, and evolutionary relationships were evaluated. This comprehensive genomic analysis explored the genetic diversity, virulence potential, and AMR profiles. Our results aid the understanding of string test-positive KP prevalence, could help guide rational drug prescription, and may enhance the control of nosocomial infections in Huzhou.

## Materials and methods

### Strain collection

In all, 632 KP isolates were collected from six sentinel hospitals of the Chinese Pathogen Identification Net of Huzhou from 2020 to 2023; the samples included sputum, urine, secretions, and blood. After suspicious colonies were isolated, species were identified using matrix-assisted laser desorption ionization time-of-flight (MALDI-TOF) mass spectrometry (bioMérieux, France). The strains were stored in glycerol broth at –70°C. *Escherichia coli* ATCC29522 was from the Chinese Center for Disease Control and Prevention.

### String test

The hypermucoviscous phenotype was identified through a positive string test. All 632 isolates were tested. After a single colony grown overnight on a Columbia blood agar plate at 36°C was stretched using an inoculation loop, the formation of a viscous string > 5 mm in length identified a hypermucoviscous strain (Kumabe and Kenzaka, 2014).

### Antimicrobial susceptibility testing

Eight classes of 17 antimicrobial agents (Thermo, USA) were used for AST. The minimum inhibitory concentrations (MICs) of chloramphenicol (CHL), trimethoprim-sulfamethoxazole (SXT), colistin (CT), ertapenem (ETP), meropenem (MEM), cefotaxime (CTX), ceftazidime (CAZ), ceftazidime/avibactam (CZA), tetracycline (TET), tigecycline (TIG), ciprofloxacin (CIP), nalidixic acid (NAL), azithromycin (AZM), amikacin (AMI), streptomycin (STR), ampicillin (AMP), and ampicillin/sulbactam (AMS) were detected using the agar dilution and broth microdilution methods. Breakpoint interpretations followed the 2020 guidelines of the Clinical and Laboratory Standards Institute (CLSI, 2020). Negative and blank controls were included; *Escherichia coli* ATCC 25922 served as a control strain.

### Whole genome sequencing

All string-positive strains were subjected to WGS. DNA was extracted from overnight cultures using QIAamp DNA Mini Kits (Qiagen, Germany) following the manufacturer’s instructions and DNA concentrations were determined using the Qubit 4 method (Thermo, USA). Quality-confirmed DNA was stored at –80°C until further use. WGS libraries were constructed using a Metagenomic DNA Library Kit (Matridx Biotechnology, China) and sequenced employing a NextSeq 550 High Output Reagent Cartridge ver. 2 (300 cycles; Illumina, USA).

### Sequence analysis

Raw sequencing data were assessed for quality, trimmed, and then assembled *de novo* into a draft genome sequence using SPAdes ver. 3.14 software. The coverage of each sequence exceeded 98% and the sequencing depth was 100X. Multilocus Sequence Typing (MLST) was performed and the K serotypes, virulence scores, plasmid status, and antimicrobial resistance genes were determined as described on the Kleborate website (https://pathogen.watch/). Kleborate is a new tool for evaluating hvKP virulence; it assigns a “virulence score” from 0 to 5. If the strain is negative for yersiniabactin, colibactin, and aerobactin, the score is 0; if it is yersiniabactin-positive only, 1; if it is yersiniabactin- and colibactin-positive (or colibactin-only positive), 2; if it is aerobactin-positive only, 3; if it is aerobactin- and yersiniabactin-positive and colibactin-negative, 4; and if it is yersiniabactin-, colibactin-, and aerobactin-positive, 5 (Lam et al., 2021).

Virulence genes were detected using the Virulent Factors of Pathogenic Bacteria (VFDB) database (http://www.mgc.ac.cn/VFs/search_VFs.htm). BioNumerics ver. 7.6 software was used to construct the minimum spanning tree. SKA was used to sort the sequencing results and obtain whole-genome single-nucleotide polymorphisms (SNPs). FastTree (version 2.1.11) software was employed for sequence alignment and homology analysis. The phylogenetic tree and heatmaps of the resistance and virulence genes were visualized using the ChiPlot tool (https://www.chiplot.online/). The genome sequences have been lodged with GenBank. The BioSample descriptor is SAMN40181878 and the accession numbers run from JBAOXF000000000 to JBAPAZ000000000.

## Results

### Isolate collection

During the 4-year screening period, 632 clinical isolates of KP from six sentinel hospitals were tested. Of these, 100 were positive in the string test (15.8%) and were further analyzed. Most isolates were from respiratory specimens (n = 57, 57.0%), followed by urine (n = 23, 23.0%), blood culture (n = 14, 14.0%), and drainage (n = 6, 5.0%). The 100 isolates were collected from 100 patients, of whom 58 (58.0%) were male. Patient age ranged from 6 to 91 years.

### Antimicrobial susceptibility profiles

The 100 string test-positive KP isolates were subjected to AST. High rates of resistance were observed for CTX (77.0%), SXT (67.0%), and NAL (64.0%); the rates for CT, TIG, and MEM were relatively low at 0.0%, 1.0%, and 3.0%, respectively. Some strains were resistant to 16 antibiotics (not CT). The resistance rates are shown in **Table 1**.

**Table 1:** Antimicrobial susceptibilities of string test-positive KP isolates.

Class	drug	susceptible		intermediate		Resistant	
		number	percentage (%)	number	percentage (%)	number	percentage (%)
β-lactam	ampicillin	1	1.0%	2	2.0%	97	97.0%
	ampicillin/sulbactam	62	62.0%	8	8.0%	30	30.0%
	ceftazidime	63	63.0%	0	0.0%	37	37.0%
	ceftazidime/avibactam	89	89.0%	2	2.0%	9	9.0%
	cefotaxime	23	23.0%	0	0.0%	77	77.0%
	ertapenem	94	94.0%	2	2.0%	4	4.0%
	meropenem	97	97.0%	0	0.0%	3	3.0%
quinolones	nalidixic acid	36	36.0%	0	0.0%	64	64.0%
	ciprofloxacin	75	75.0%	5	5.0%	20	20.0%
macrolides	azithromycin	86	86.0%	0	0.0%	14	14.0%
aminoglycosides	streptomycin	88	88.0%	4	4.0%	8	8.0%
	amikacin	89	89.0%	2	2.0%	9	9.0%
tetracyclines	tetracycline	82	82.0%	6	6.0%	12	12.0%
	tigecycline	99	99.0%	0	0.0%	1	1.0%
Phenylpropanols	chloramphenicol	31	31.0%	8	8.0%	61	61.0%
sulfonamides	trimethoprim-sulfamethoxazole	33	33.0%	0	0.0%	67	67.0%
lipopeptides	colistin	100	100.0%	0	0.0%	0	0.0%

### MLST and K-serotype

**Figure 1:**
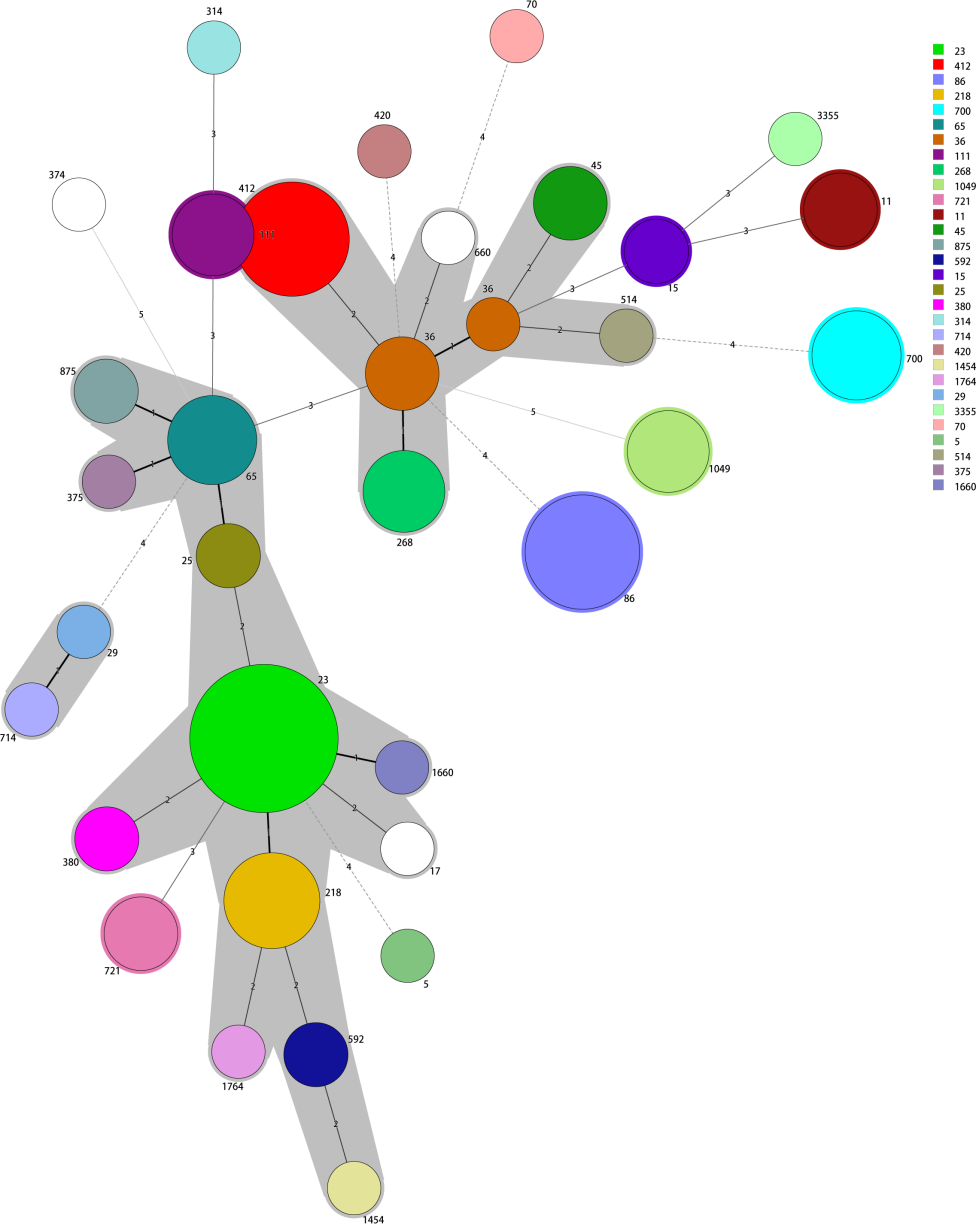
The minimum spanning tree reveals potential relationships among isolates of different sequence types (STs). The numbers on the branches represent the differences in the seven housekeeping genes that determine the STs. ST23 is the most common type; most other STs differ from ST23 in fewer than two housekeeping genes.

MLST identified 33 sequence types among the positive KPs, principally ST23, ST86, ST412, ST218, ST65, ST700, ST36, ST111, ST268, and ST1049. Of these, ST23 accounted for 16.0% (16/100), followed by ST86 (9.0%, 9/100) and ST412 (9.0%, 9/100). In all, 15 K serotypes were detected, with two strains having unknown serotypes. The dominant serotype was K1 (22.5% [22/98]), followed by K2 and K57 (20.4% [20/98] and 17.4% [17/98], respectively). The K serotypes were generally consistent within the same ST strains, thus ST23-K1, ST86-K2, ST412-K57, ST218-K57, and ST65-K2. However, the two ST15 strains exhibited different K-serotypes, K19 and K24. The minimum spanning tree is shown in **Figure 1**.

### Antimicrobial-resistance genes

**Figure 2:**
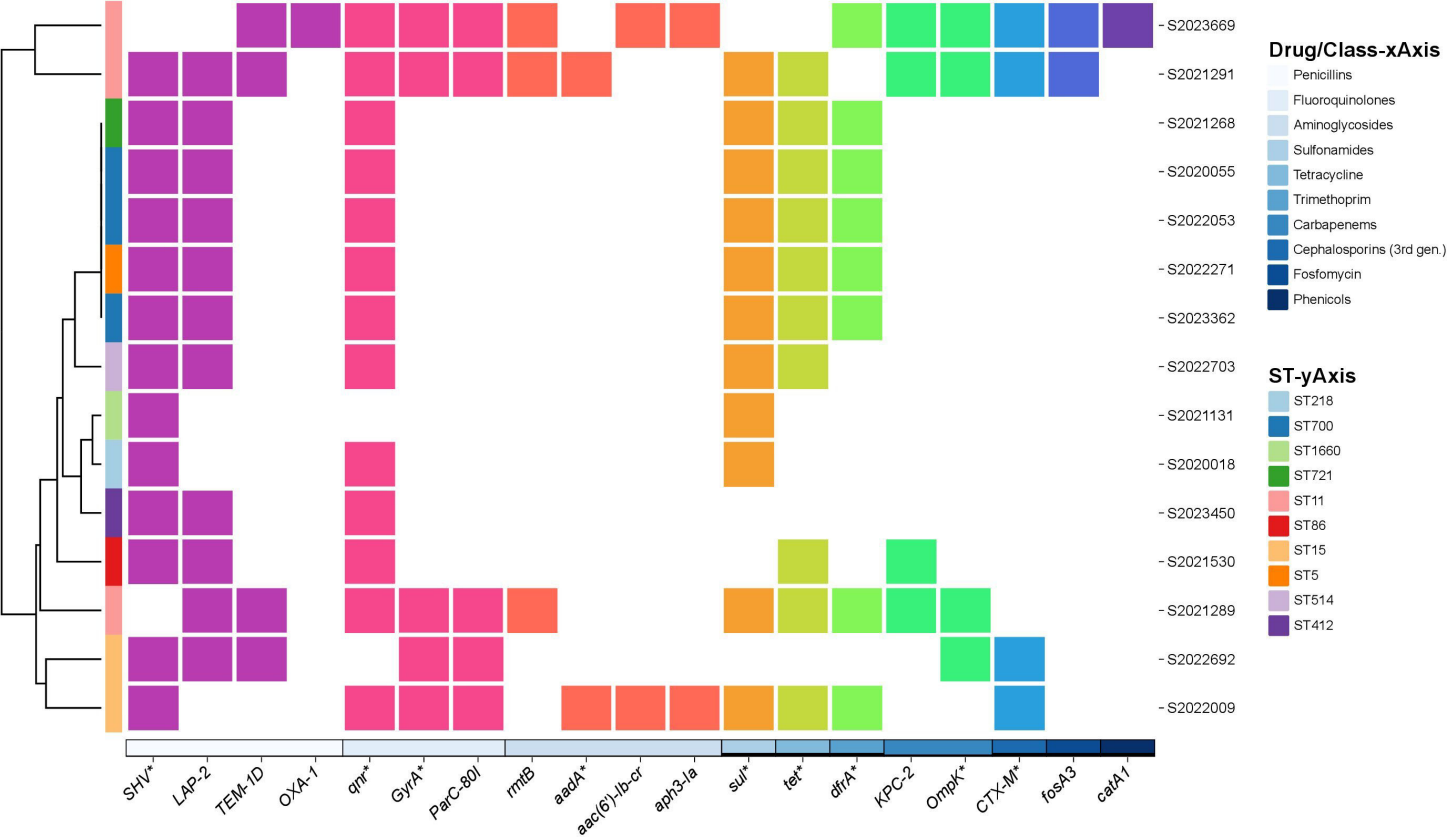
Annotation heatmap of antimicrobial resistance genes among 15 string test-positive KP strains with more than two resistance genes isolated in Huzhou from 2020 to 2023.

We identified 19 drug-resistance genes of 10 classes. The *SHV* point mutation, linked to penicillin resistance, was the most common (98.0% of isolates); only two strains lacked *SHV* mutations. In the 98 strains with *SHV* variants, *SHV-11*, *SHV-1*, and *SHV-142* were the most prevalent. Eighty-five strains had single *SHV*-resistance genes but 15 had more than two genes. That are shown in **Figure 2**. Resistance to penicillin mediated by *SHV*, *LAP-2*, *TEM-1D*, and *OXA-1* mutations was observed in 98.0%, 11.0%, 4.0%, and 1.0% of isolates, respectively. Genes mediating resistance to aminoglycosides, quinolones, carbapenems, sulfonamides, TET, trimethoprim, cephalosporins (third generation), fosfomycin, and phenicols were identified. Antimicrobial resistance genes among 98 string test-positive KP strains are illustrated in **Supplementary Figure 1**.

### Virulence factors and scores

**Figure 3:**
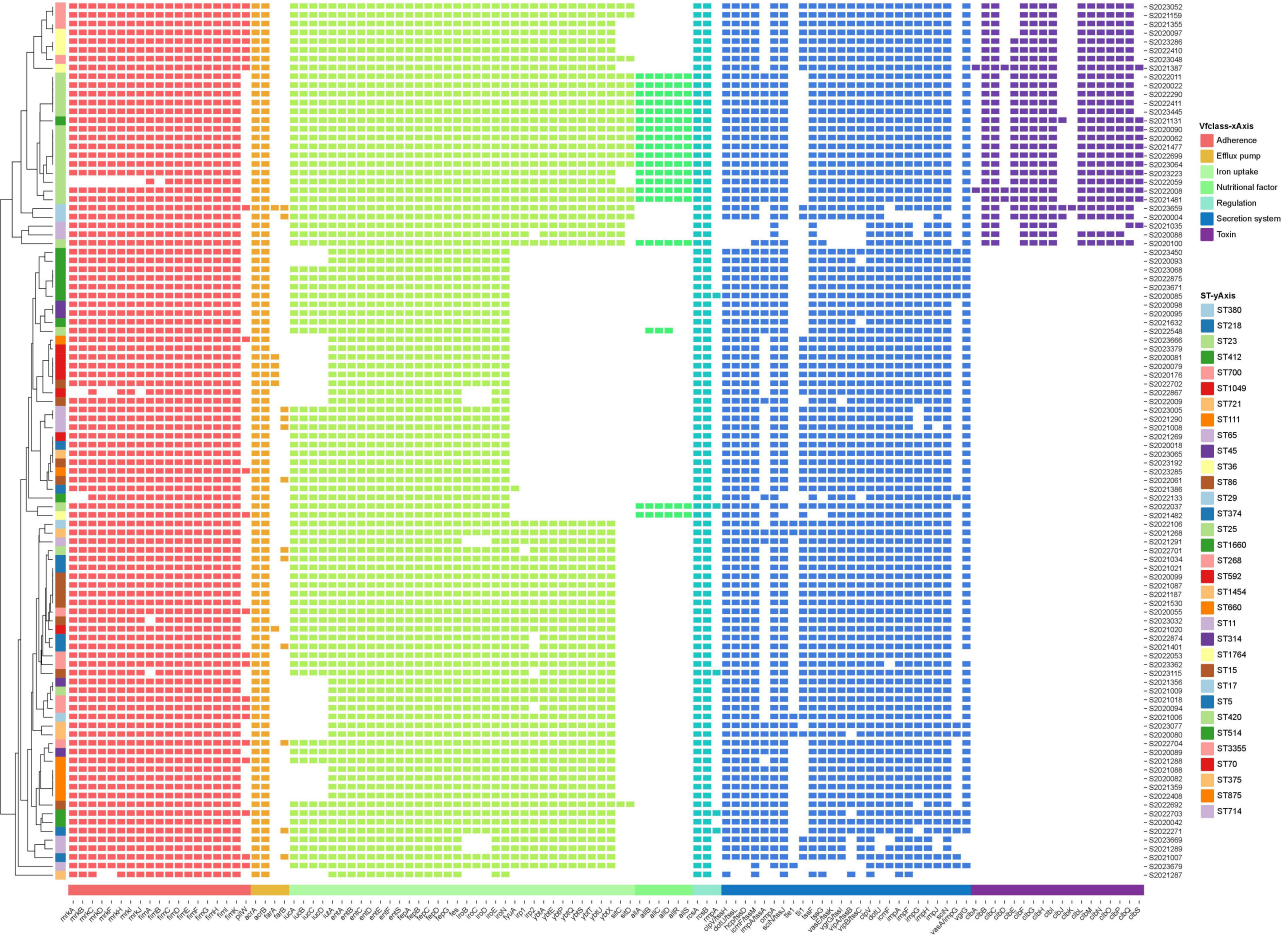
Annotation heatmap of virulence genes among 100 string test-positive KP strains in Huzhou from 2020 to 2023.

Virulence analysis using the VFDB database identified 112 virulence genes among the string test-positive KP isolates; Vfclass divided these into seven groups based on their roles in pathogenesis. Adherence genes included type 3 fimbriae (*mrkA*, *mrkB*, *mrkB*, etc.), type I fimbriae (*fimA*, *fimB*, *fimC*, etc.), and type IV pili (*pilW*). Efflux pump genes included *acrAB* (*acrA*, *acrB*) and *farAB* (*farA*, *farB*). Iron uptake was affected by several virulence factors including mutations in aerobactin (*iucA*, *iucB*, *iucC*, etc.), the *ent* siderophore (*entA*, *entB*, *entC*, etc.), salmochelin (*IroB*, *IroC*, *IroD*, etc.), yersiniabactin (*fyuA*, *irp1*, *irp2*, etc.), and iron transport genes (*sitC* and *sitD*). Virulence factors affecting nutrition and regulation included mutations in genes of allantoin utilization (*allA*, *allB*, *allC*, etc.) and *rcsAB* (*rcsA*, *rcsB*, *rmpA*), respectively. The secretion system was affected by mutations in the T6SS-I (*ompA*, *tle1*, *tli1*, etc.) and T6SS-II/III (*dotU*, *icmF*, *impF*, etc.) clusters. The toxin genes included colibactin virulence factors (*clbA*, *clbB*, *clbC*, etc.). The virulence genes are illustrated in **Figure 3**.

The Kleborate virulence scores differed somewhat; not all 100 strains were hypervirulent. Twenty-eight strains scored 5, 28 scored 4, 21 scored 3, 12 scored 1, and 11 scored 0. The STs associated with virulence scores of 5 were ST23, ST380, ST268, ST36, and ST65.

### Plasmids

**Figure 4:**
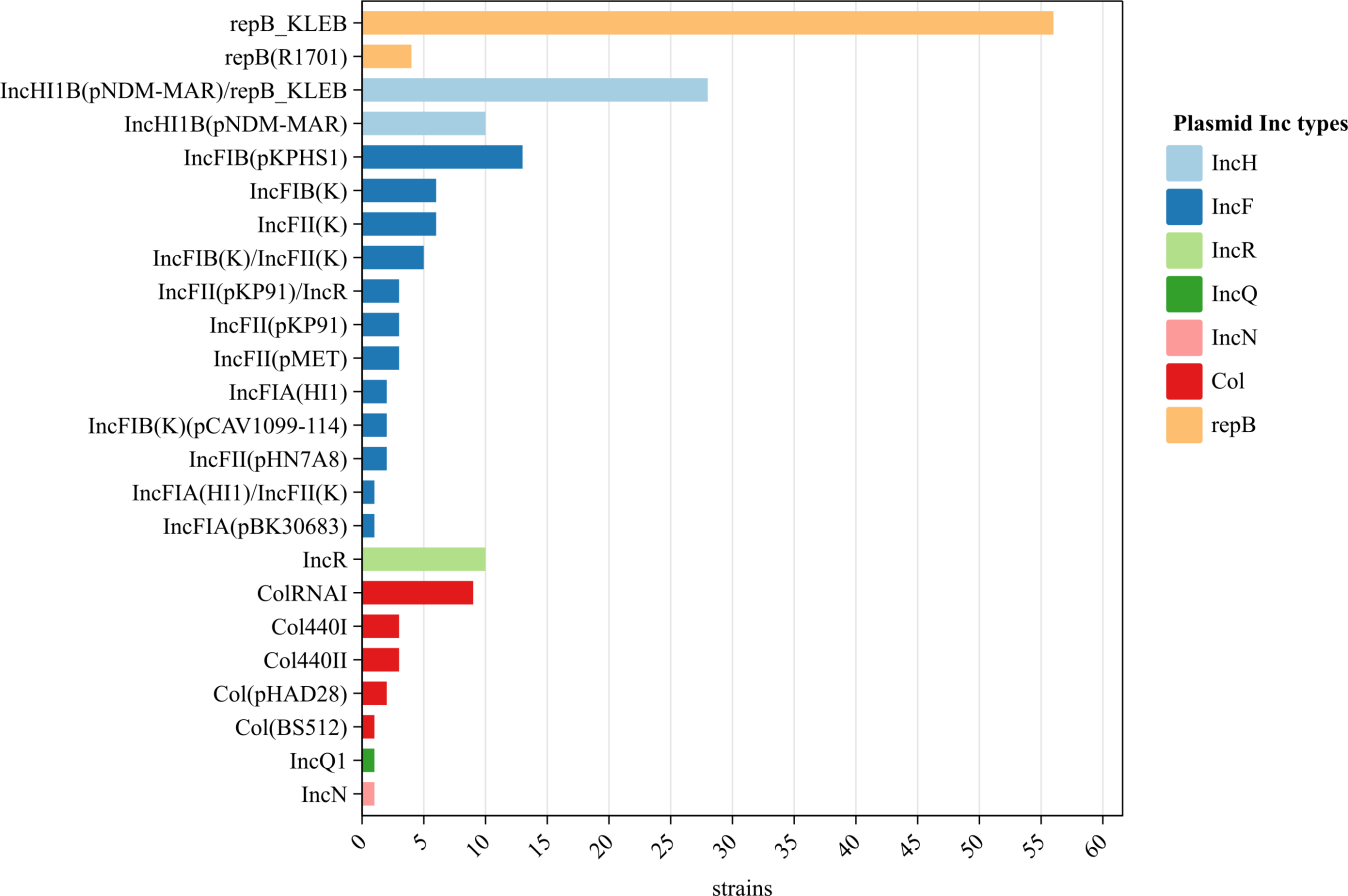
Plasmids of the 100 string test-positive KP strains in Huzhou from 2020 to 2023.

No plasmids were detected in two strains but 24 plasmids (of seven families) were identified in the remaining 98 strains. The IncF plasmid was the most diverse (11 subtypes). Of the 24 plasmids, repB_KLEB (57.1%, 56/98), IncHI1B(pNDM-MAR)/repB_KLEB (28.6%, 28/98), and IncFIB(pKPHS1) (13.3%, 13/98) were the most common; the occurrence rates of other plasmids ranged from 1.0% to 10.2% (**Figure 4**).

### Phylogenetic analysis

**Figure 5:**
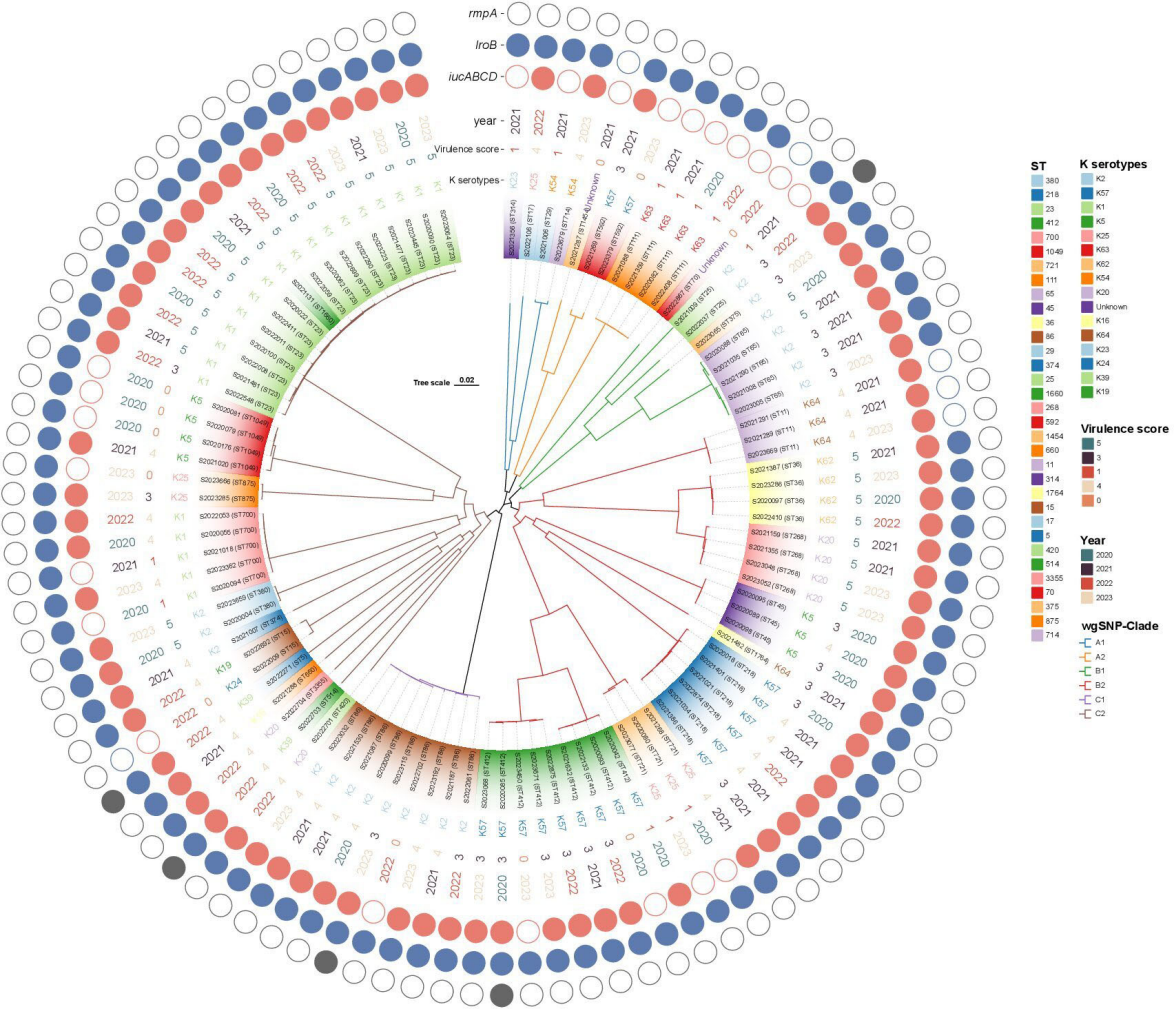
Phylogenetic analysis of 100 string test-positive KP isolates in Huzhou from 2020 to 2023.

WgSNP phylogenetic analysis of the 100 string test-positive KP isolates identified three major clades designated A–C (**Figure 5**), but no association between any lineage and the year of isolation. Each clade included two subclusters (A1-A2, B1–B2, C1–C2). Clade A1 contained four strains (ST314, ST17, ST27, and ST714) and clade A2 had seven (ST1454, ST592, and ST111). Clades B1 and B2 contained 9 and 33 strains, respectively. The B1 ST types were ST70, ST25, ST375, and ST65. The B2 ST types were ST11, ST36, ST268, ST45, ST1764, ST218, ST721, and ST412. Clade C1 exhibited only one ST type, ST86 (nine strains). The largest clade, clade C2, included 38 isolates with diverse ST types as follows: ST420, ST514, ST3355, ST660, ST5, ST15, ST374, ST380, ST700, ST875, ST1049, ST1660, and ST23.

## Discussion

The latest data from the China Antimicrobial Resistance Surveillance System (CARSS) indicate that the KP clinical isolation rate is 21.2% of all Gram-negative bacteria, thus second to that of *E*. *coli*; KP is one of the most common causes of clinical infections in China. hvKP infections occur at unusual (sometimes multiple) sites, often accompanied by bacteremia and metastatic spread (Prokesch et al., 2016; Li et al., 2018; Wyres et al., 2020). To investigate the molecular epidemiological characteristics of hvKP in Huzhou, 100 KPs that were positive in string-tests were collected from 632 isolates, evaluated in terms of antimicrobial susceptibility, and subjected to WGS. Of these, 100 (15.82%) were string-test positive, fewer than the 32.03% in Guizhou province reported by Zhou et al. (2021) but more than the 7.63% in Germany reported by Neumann et al. (2023). The variation may be attributable to differences in economic or medical conditions. Respiratory specimens were the major source of positive isolates in Huzhou; patients with such infections require careful attention.

Antimicrobial resistance has long compromised human health. The misuse of antibiotics in clinical practice, agriculture, and aquaculture has exacerbated the problem (Arias and Murray, 2009). hvKP antimicrobial resistance can arise via acquisition of a plasmid carrying resistance genes, chromosomal gene mutations, or the transfer of hypervirulent plasmids to multidrug-resistant strains (Dong et al., 2018; Feng et al., 2018; Gu et al., 2018). All hvKP strains exhibit an inherent resistance to AMP; we observed a high resistance rate (Russo and Marr, 2019). Of the 97 AMP-resistant strains, 100.0% (n = 97) harbored the *SHV* gene. High resistance rates were also observed for NAL, CHL, and SXT, consistent with previous findings (Wang et al., 2022). Caution should be exercised when using these antibiotics to treat hvKP infections. Notably, we found no CT-resistant strain. CT is very active against most Gram-negative bacteria and is often considered the “last resort” treatment for multidrug-resistant bacteria (Biswas et al., 2012).

Genomic studies have shown that KP (including hvKP) genomes are diverse but highly structured (Wyres et al., 2020). Our 100 isolates exhibited the hypermucoviscous phenotype (positive string test results) and were of 33 ST types with 15 capsular serotypes. Notably, the K1 capsular type was the predominant serogroup responsible for KP infections in Huzhou; most K1-type strains were of the ST23 type, as reported by Tang et al. (2020) and Hallal Ferreira Raro et al. (2023). The most common capsular locus associated with positive KPs was K1 (22.5%), followed by K2 (20.4%), K57 (17.4%), and K5 (7.1%), consistent with other regional studies (Lee et al., 2016; Guo et al., 2017). Most positive strains of the K1 serotype were of type ST23, but K2 serotype strains were of several ST types including ST86, ST65, and ST25, as in previous studies (Struve et al., 2015; Anantharajah et al., 2022). In addition, we report a K20 ST3355 isolate. New hvKP STs continue to emerge, indicating ongoing evolution (Lam et al., 2018; Wyres et al., 2020). The minimum spanning tree divided the 100 strains into two evolutionary branches with ST23 and ST412 as the cores.

hvKP is inherently resistant to AMP because a β-lactamase enzyme is encoded by chromosomal *SHV*. Of our strains, 98 with *SHV* genes exhibited wild-type *in vitro* susceptibility to β-lactams. Of these, 85 carried only one *SHV* resistance gene, but the remaining 15 had more than two, including genes conferring resistance to various antibiotics such as penicillin (*LAP-2*, *TEM-1D*, *OXA-1*), aminoglycosides (*rmtB*, *aadA*, *aac(6’)-Ib-cr* and *aph3-Ia*), fluoroquinolones (*qnr*, *GyrA*, and *ParC-80I*), carbapenems (*OmpK* and *KPC-2*), sulfonamides (*sul*), TET (*tet*), trimethoprim (*dfrA*), cephalosporins (*CTX-M*), fosfomycin (*fosA3*), and phenicols (*catA1*). Specific mutations in the outer membrane porin *OmpK* gene contribute substantially to the carbapenem-resistance of hvKP. Porin defects can increase the MIC to above the levels conferred by acquired carbapenemase genes alone (Fajardo-Lubián et al., 2019). The *fosA3* gene of hvKP reduces fosfomycin susceptibility to below the breakpoint of clinically relevant resistance. ESBLs (encoded by *CTX-M*, *SHV*, and *TEM*, and so forth) are modified broad-spectrum β-lactamases that hydrolyze third-generation cephalosporins, aztreonam, and fourth-generation cephalosporins. hvKPs that express ESBLs may also possess porin mutations that decrease the uptake of cephalosporins and carbapenems, further reducing susceptibility to such agents (Surgers et al., 2016; Yu et al., 2017). Of the four strains exhibiting ETP-resistant phenotypes, all carried the *KPC-2* carbapenem-resistance gene. *KPC* transposon-mediated spread is increasingly reported globally. Such spread is frequently coupled to the dissemination of other β-lactamases (Xu et al., 2019); here, we describe the coexistence of a *KPC* gene and *ESBL-*encoding genes.

Although many studies have defined hvKP as a positive result in the string test, not all hvKP strains are hypermucoviscous (Fu et al., 2018; Russo and Marr, 2019). The predominant aerobactin siderophore enhances virulence, and it is likely to be a reliable biomarker of hvKP strains (Russo et al., 2015). Russo et al. (2018) reported that *iucA* of the *iuc* operon encoding aerobactin very accurately differentiated hvKP from cKP strains; *iucA-*positivity indicated hypervirulence. As shown in **Figures 3**, **5**, 78.0% (78/100) of strains carried *iucA*; KP hypervirulence can thus be preliminarily indicated by a positive string test. The status of *iroB* (encoding an enzyme of salmochelin siderophore biosynthesis) is also important when determining whether a strain is hypervirulent (Russo et al., 2018); the carrying rate of *iroB* was 94.0% (94/100) in our study. The biosynthesis of salmochelin and aerobactin is encoded by the *iroB* and *iucA* clusters of virulence plasmids, respectively. *RmpA* is a plasmid-located virulence factor that regulates the synthesis of capsular polysaccharides (Wang et al., 2020). Lin et al. (2020) found that *rmpA* deletion eliminated the hypermucoviscous phenotype and decreased virulence; *rmpA* overexpression increased virulence. Although *rmpA* has been considered a useful genetic marker of hvKP, only five of our strains carried it. Thus, although *rmpA* increases virulence, it may not be the optimal indicator of hypervirulence (Neumann et al., 2023).

Not all KP strains that are positive in string tests exhibit high virulence scores (Russo et al., 2018); 11 of our strains had virulence scores of 0 and were thus hypermucoviscous cKP (non-hypervirulent) strains. As shown in **Figure 5**, virulence was not closely associated with the ST type or serotype but rather with *iucA* gene-positivity. The *iucA* gene was not detected in 11 strains, but of these, 8 (72.7%) carried the *iroB* gene. Of the 28 strains that scored 5 in the virulence test, ST23-K1 accounted for 53.6% (15/28), ST36-K62 for 14.3% (4/28), and ST268-K20 for 14.3% (4/28). Interestingly, patient age was associated with virulence scores; 78.6% (n = 22) of strains with virulence scores of 5 (n = 28) were from patients over 60 years of age, perhaps reflecting poor immune function in the elderly. The *iucA* gene was detected in all 77 strains with virulence scores of 3, 4, and 5. Thus, *iucA*-positivity well-reflects hypervirulence status.

The widespread IncF plasmid of Enterobacteriaceae typically carries β-lactamase and carbapenemase genes that significantly contribute to KP drug resistance. The IncR plasmid is predominantly found in *Salmonella* and can harbor various drug-resistance genes associated with multiple drug-resistance phenotypes in human hosts. This plasmid can be horizontally transferred between bacterial strains (Qian et al., 2020).

String test-positive KP strains in Huzhou have been understudied. We used wgSNP phylogenetic analysis to show that strains from patients in Huzhou could be categorized into six main clades (A1, A2, B1, B2, C1, and C2). Strains sharing the same STs were more closely related and tended to carry the same virulence and antibiotic-resistance genes. Interestingly, closely related strains were found in both the same and different hospitals, and in the same and different years. Associations between clades and certain resistance and virulence determinants were common, although not all clades of a phylogenetic tree exhibited particular geographic distributions or years of isolation.

Physicians who encounter invasive infections caused by KP should prioritize prompt microbiological tests that differentiate hvKP from cKP via molecular characterization of the major virulence factors. Virulence genes commonly found in hvKP strains (e.g., *iucA*) might serve as genotypic biomarkers for early detection of hvKP in areas of low KP prevalence. Today, hvKP poses a significant public health threat; rigorous monitoring is essential to prevent widespread transmission and potential infectious outbreaks.

Our work had several limitations. First, a positive result in the string test does not inevitably indicate hypervirulence. Catalán-Nájera et al. (2017) noted that not all hvKPs exhibit a hypermucoviscous phenotype; thus, we may have missed some hvKPs during screening. Second, we lacked details on the infections and treatment results; we do not know what harm the patients suffered.

## Conclusions

We present an extensive genomic analysis of KPs isolated in Huzhou that tested positive in the string test. This affords valuable insights into ST prevalences, capsular serotypes, antimicrobial-resistance markers, virulence genes, and phylogenetic relationships from 2020 to 2023. The rates of resistance to CTX, SXT, and NAL were high but resistance to CT was low. The *iucA* gene of hvKP may serve as a genotypic biomarker for early detection. It is essential to develop monitoring, control, and prevention strategies that address the increasing threats posed by hvKP.

## Data availability statement

The datasets presented in this study can be found in online repositories. The names of the repository/repositories and accession number(s) can be found in the article/**Supplementary Material**.

## Ethics statement

The studies involving humans were approved by the human research ethics committee of the Huzhou Center for Disease Control and Prevention. The studies were conducted in accordance with the local legislation and institutional requirements. Written informed consent for participation in this study was provided by the participants’ legal guardians/next of kin.

## Author contributions

WY: Conceptualization, Data curation, Formal analysis, Writing – original draft, Writing – review & editing. DX: Investigation, Methodology, Writing – review & editing. YS: Validation, Writing – original draft. FD: Software, Writing – original draft. LJ: Writing – review & editing.
