# Supplementary figures and images for: Molecular epidemiology of string test-positive Klebsiella pneumoniae isolates in Huzhou, China, 2020-2023
Source: Front Cell Infect Microbiol. 2024 Aug 6;14:1411658. doi: 10.3389/fcimb.2024.1411658 (PMC11333340; doi:10.3389/fcimb.2024.1411658)

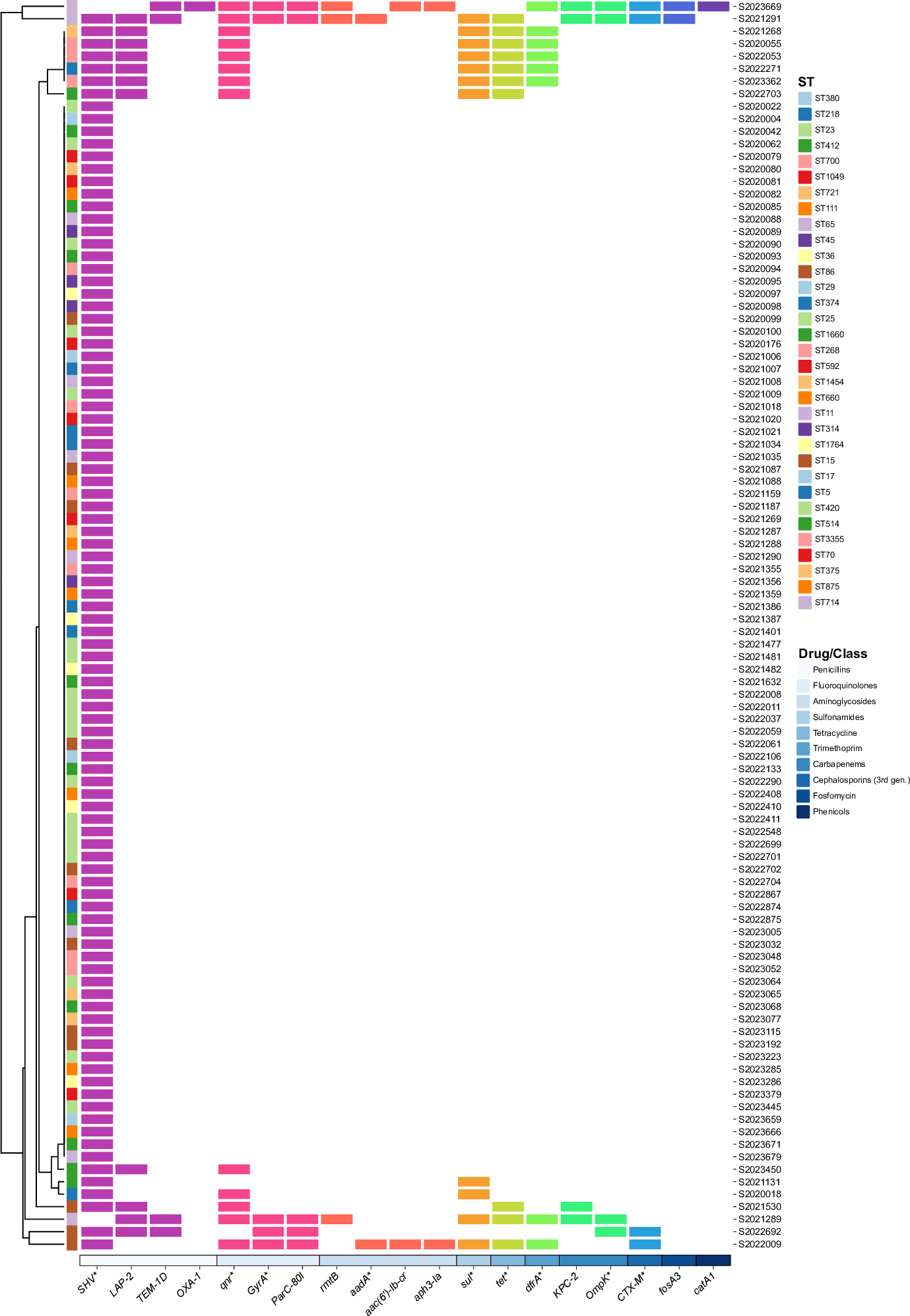

Supplement: Supplementary file 1 [file Image_1.tif]
